# Screening mammography performance according to breast density: a comparison between radiologists versus standalone intelligence detection

**DOI:** 10.1186/s13058-024-01821-w

**Published:** 2024-04-22

**Authors:** Mi-ri Kwon, Yoosoo Chang, Soo-Youn Ham, Yoosun Cho, Eun Young Kim, Jeonggyu Kang, Eun Kyung Park, Ki Hwan Kim, Minjeong Kim, Tae Soo Kim, Hyeonsoo Lee, Ria Kwon, Ga-Young Lim, Hye Rin Choi, JunHyeok Choi, Shin Ho Kook, Seungho Ryu

**Affiliations:** 1 Department of Radiology, Kangbuk Samsung Hospital, Sungkyunkwan University School of Medicine, Seoul, South Korea; 2 Center for Cohort Studies, Kangbuk Samsung Hospital, Sungkyunkwan University School of Medicine, Samsung Main Building B2, 250, Taepyung-ro 2ga, Jung-gu, 04514 Seoul, South Korea; 3 Department of Occupational and Environmental Medicine, Kangbuk Samsung Hospital, Sungkyunkwan University School of Medicine, Seoul, Republic of Korea; 4 Department of Clinical Research Design & Evaluation, Samsung Advanced Institute for Health Sciences & Technology, Sungkyunkwan University, Seoul, Republic of Korea; 5 Department of Surgery, Kangbuk Samsung Hospital, Sungkyunkwan University School of Medicine, Seoul, Republic of Korea; 6 Lunit Inc, Seoul, Republic of Korea; 7 Department of Statistics, Ewha Womans University, Seoul, Republic of Korea; 8 Institute of Medical Research, Sungkyunkwan University School of Medicine, Suwon, Republic of Korea; 9 School of Mechanical Engineering, Sunkyungkwan University, Seoul, Republic of Korea

**Keywords:** Mammography, Breast, Screening, Intelligence, Asian women

## Abstract

**Background:**

Artificial intelligence (AI) algorithms for the independent assessment of screening mammograms have not been well established in a large screening cohort of Asian women. We compared the performance of screening digital mammography considering breast density, between radiologists and AI standalone detection among Korean women.

**Methods:**

We retrospectively included 89,855 Korean women who underwent their initial screening digital mammography from 2009 to 2020. Breast cancer within 12 months of the screening mammography was the reference standard, according to the National Cancer Registry. Lunit software was used to determine the probability of malignancy scores, with a cutoff of 10% for breast cancer detection. The AI’s performance was compared with that of the final Breast Imaging Reporting and Data System category, as recorded by breast radiologists. Breast density was classified into four categories (A–D) based on the radiologist and AI-based assessments. The performance metrics (cancer detection rate [CDR], sensitivity, specificity, positive predictive value [PPV], recall rate, and area under the receiver operating characteristic curve [AUC]) were compared across breast density categories.

**Results:**

Mean participant age was 43.5 ± 8.7 years; 143 breast cancer cases were identified within 12 months. The CDRs (1.1/1000 examination) and sensitivity values showed no significant differences between radiologist and AI-based results (69.9% [95% confidence interval [CI], 61.7–77.3] vs. 67.1% [95% CI, 58.8–74.8]). However, the AI algorithm showed better specificity (93.0% [95% CI, 92.9–93.2] vs. 77.6% [95% CI, 61.7–77.9]), PPV (1.5% [95% CI, 1.2–1.9] vs. 0.5% [95% CI, 0.4–0.6]), recall rate (7.1% [95% CI, 6.9–7.2] vs. 22.5% [95% CI, 22.2–22.7]), and AUC values (0.8 [95% CI, 0.76–0.84] vs. 0.74 [95% CI, 0.7–0.78]) (all *P* < 0.05). Radiologist and AI-based results showed the best performance in the non-dense category; the CDR and sensitivity were higher for radiologists in the heterogeneously dense category (*P* = 0.059). However, the specificity, PPV, and recall rate consistently favored AI-based results across all categories, including the extremely dense category.

**Conclusions:**

AI-based software showed slightly lower sensitivity, although the difference was not statistically significant. However, it outperformed radiologists in recall rate, specificity, PPV, and AUC, with disparities most prominent in extremely dense breast tissue.

**Supplementary Information:**

The online version contains supplementary material available at 10.1186/s13058-024-01821-w.

## Background

Mammography serves as the primary screening method for breast cancer and has significantly reduced breast cancer mortality rates by approximately 40%, with annual screenings starting at 40 years old [1]. Despite its effectiveness, the quality of its assessment varies among radiologists, and breast cancer can be missed due to detection or misinterpretation errors [2, 3]. Additionally, mammography screening may have limitations for some women, especially women with dense breasts; sensitivity values range from 47 to 62% for extremely dense breasts [4, 5]. The false-positive mammography rate for dense breasts is higher than that for non-dense breasts [6, 7]. Thus, enhancing the accuracy of screening mammography in women with dense breasts is crucial for addressing this issue.

Conventional computer aided diagnosis (CAD) was introduced as a secondary diagnostic tool for radiologists to improve the performance of screening mammography [8, 9]. CAD has lower specificity owing to numerous false-positive CAD markers without resulting in significantly increased sensitivity. Recently, artificial intelligence (AI)-driven CAD, fueled by deep learning and convolutional neural networks, has been developed to increase accuracy and reduce performance variations among radiologists [10–15]. Assistance of AI algorithms has significantly improved the overall performance of radiologists [10–12, 16, 17].

While the use of AI as a stand-alone reader of mammograms can enhance the workload efficiency of screening programs, for AI to truly improve screening outcomes and workload efficiency, its stand-alone performance should be sufficiently high. One recent meta-analysis by Yoon et al., evaluating more than one million mammograms, found that areas under the receiver operating curve (AUCs) were significantly higher for standalone AI than radiologists in reader studies involving cancer-enriched populations, but not in historic cohort studies [18].

Compared with Western women, Asian women usually have higher breast density (> 50% have dense breast tissue), which is an independent risk factor for breast cancer [19, 20]. Their unique characteristics include smaller breasts, lean body mass, and distinct breast cancer features [21] Previous cohort studies of standalone AI were primarily conducted on Western populations [15, 22–26], and few large studies have evaluated the standalone AI in real screening settings involving Asian women, particularly those with dense breast tissue. Enhancing breast cancer screening effectiveness in Asian populations with dense breasts can be facilitated by achieving favorable screening outcomes using AI algorithms.

Therefore, this study investigated the performance metrics of screening digital mammography by comparing radiologists results with those of standalone AI detection in a screening cohort of East Asian women, considering breast density.

## Methods

### Study population

The Kangbuk Samsung Health Study is a cohort study of Korean men and women aged ≥ 18 years who underwent comprehensive annual or biennial health examinations at Kangbuk Samsung Hospital Total Healthcare Centers in Seoul and Suwon, South Korea, as previously described [27, 28]. This study was approved by the Institutional Review Board of Kagnbuk Samsung Hospital (approval number: 2020-11-010), which waived the requirement for informed consent owing to the use of de-identified retrospective data collected during the health screening process.

**Fig. 1:**
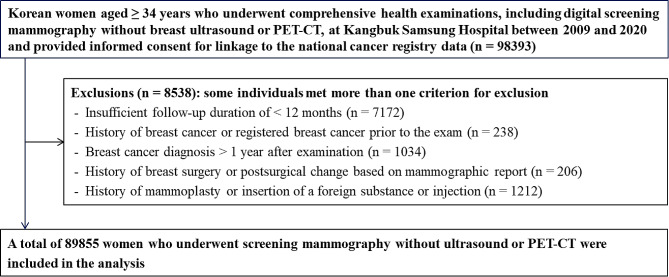
Flowchart of the study population. PET-CT, positron emission tomography-computed tomography

This retrospective study focused on Korean women aged ≥ 34 years who underwent initial digital screening mammography at our institution as part of a health examination between January 2009 and December 2020 (Fig. 1). Participants who underwent simultaneous breast ultrasonography and positron emission tomography-computed tomography examinations were excluded. Only participants who provided informed consent for linkage of their data to the national cancer registry data were included in the study. Notably, while national guidelines in Korea recommend breast cancer screening starting at the age of 40 years, private screening organizations commonly offer screenings from the age of 35 years [29, 30]. Considering the distinction between the recommended screening ages in Korea and Western countries, which can lead to a difference of 1–2 years in the recorded data, we included patients who were actually 34 years old, supported by real data. Participants with follow-up durations of < 12 months from the end of the cancer registry date (December 31, 2020), a history of breast cancer or a prior registered breast cancer before mammography, a breast cancer diagnosis > 1 year after screening mammography, a history of breast surgery or postsurgical changes based on mammographic reports, or mammographic findings indicating mammoplasty or foreign substance insertion or injection were excluded. After applying the exclusion criteria, 89,855 women were included in the final analysis.

### Data collection

Demographic information, first-degree family history of breast cancer, behavioral factors, reproductive factors, and medical history, including history of benign breast disease, were collected using standardized, self-administered questionnaires. Trained nurses measured body height and weight with the participant wearing a hospital gown and bare feet. Body mass index (BMI) was then classified according to the Asian-specific criteria [31] as follows: underweight, < 18.5 kg/m^2^; normal weight, 18.5–23 kg/m^2^; overweight, 23–25 kg/m^2^; and obese, ≥ 25 kg/m^2^.

### Mammography acquisition and assessment

Mammographic imaging data, encompassing Breast Imaging Reporting and Data System (BI-RADS) categories and mammographic density, were extracted from the original radiological reports. The study participants underwent standard four-view digital mammography, comprised of bilateral craniocaudal (CC) and mediolateral oblique (MLO) views, using a full-field digital mammography system (Senographe 2000D/DMR/DS [GE Healthcare, Chicago, IL, USA] or Selenia [Hologic, Marlborough, MA, USA]) at the Suwon and Seoul Total Healthcare Centers. In this study, 97.6% of mammograms were captured using Senographe 2000D/DMR/DS systems [GE Healthcare, Chicago, IL, USA]. Starting from 2016, our institution implemented the Selenia system [Hologic, Marlborough, MA, USA], predominantly for tomosynthesis during the initial two years, rather than routine mammography. The final mammography assessment was conducted by one of six experienced breast imaging radiologists at one of the two centers using the BI-RADS classification system [32]. This system rates findings on a scale from 0 to 5, which reflects the degree of suspicion of malignancy as follows: negative (1), benign (2), probably benign (3), needs additional imaging evaluation (0), suspicious (4), and highly suggestive of malignancy (5). Breast density was visually assessed by radiologists and categorized based on the BI-RADS assessment as types A (almost entirely fatty), B (scattered fibroglandular densities), C (heterogeneously dense), or D (extremely dense).

In this study, an AI algorithm (Lunit Inc., INSIGHT MMG, version 1.1.7.2) was applied retrospectively to the stored mammographic images. The AI algorithm, a diagnostic support software that aids in mammogram reading by detecting breast cancer, was developed using a deep convolutional neural networks approach. The neural network of the AI algorithm consists of two components: a feature extractor backbone based on a ResNet-34 [33] implementation and task-specific modules for predicting cancer and density. It was developed and validated with more than 170,000 mammographic examinations obtained from three institutions in South Korea, one institution in the United States, and one in the United Kingdom [10]. The mammograms used for development and validation were done using different equipment, including GE, Hologic and Siemens systems.

To process large-scale mammography data more efficiently, we processed all cases in parallel using the AI model implementation. We note that although the inference scheme differs from that in similar studies performed using a commercial product or validator tool, the output of the AI model is equivalent regardless of the inference scheme.

The AI algorithm consists of two components: the cancer module produces pixel-level abnormality scores and a view-level abnormality score is determined by a maximum of the per-view pixel-level outputs. Abnormality scores ranged from 0−100%. The AI algorithm outputs breast-level abnormality scores by finding the maximum of abnormality scores of CC and MLO view-level scores. The density task module shares a common feature extractor with the cancer module and outputs a discrete score for density prediction, ranging from 1−10. We computed a density estimation for each patient by extracting the density score for each view and then calculating the averages across all CC and MLO views.

The AI results were categorized as test-positive if a cancer probability score of ≥ 10% was detected in either breast; otherwise, the results were classified as negative [10, 22, 34]. The average mammographic density was presented on a scale from 1 to 10, with the density categories defined as follows: Density A (scores 1−2), Density B (scores 3−5), Density C (scores 6−8), and Density D (scores 9−10) [35].

### Ascertainment of breast cancer

The reference standard for determining the presence or absence of a breast cancer diagnosis within 12 months after a screening mammography was established by linking the study data to the Korean Central Cancer Registry [36]. Breast cancer was defined as invasive cancer (International Classification of Diseases-10 code C50) or ductal carcinoma in situ (International Classification of Diseases-10 code D05.1). Tumor stages were retrieved from the registry and recorded as localized, regional, distant, or unknown, according to the National Cancer Institute Surveillance, Epidemiology, and End Results categories. Furthermore, data on treatments administered within the first 4 months from the date of the initial breast cancer diagnosis were also obtained for analysis.

### Statistical analyses

The final BI-RADS categories and breast density on screening mammography were determined from the original radiological reports. Radiological reports categorized as BI-RADS 0, 3, 4, or 5 were considered test-positive for malignancy, whereas BI-RADS 1 or 2 were classified as test-negative.

The screening digital mammography performance metrics were evaluated overall and across breast density categories and compared between radiologists and AI standalone detection. Performance indicators, including the cancer detection rate (CDR), sensitivity, specificity, positive predictive value (PPV), and recall rate, were assessed. The CDR was calculated as the number of detected cancers per 1000 examinations. Sensitivity was determined as the ratio of positive examinations with tissue-diagnosed cancer within 12 months to the total number of cancers in the cohort, whereas specificity was defined as the ratio of negative examinations without tissue-diagnosed cancer within 12 months to the total number of cancer-free examinations. Interval cancer was defined as cancer diagnosed within the 12-month follow-up period after a negative screening mammogram. The PPV was defined as the percentage of positive examinations resulting in tissue-diagnosed cancer within 12 months of screening. The recall rate was defined as the percentage of positive examinations among the total screening examinations. The 95% confidence intervals (CIs) were calculated. Additionally, the performance of screening mammography to predict a breast cancer diagnosis according to the national cancer registry data was evaluated using the AUC. The differences in AUC values between radiologists and AI standalone detection were assessed using the *roccomp* command in Stata software. Otherwise, McNemar’s test was used to analyze the statistical differences among the performance metrics.

To conduct a stratified analysis by breast density, breast density was classified into the following categories: types A and B (non-dense), type C (heterogeneously dense), or type D (extremely dense). For this analysis, both the radiologist reports and AI categories were used as valuable indicators of breast density. Logistic regression with the generalized estimating equation method was used to compare cancers detected by the AI algorithm and by radiologists.

All analyses were performed using Stata software (version 17.0; StataCorp LLC, College Station, TX, USA). Statistical significance was defined as a two-tailed P-value of < 0.05.

## Results

### Baseline characteristics

A total of 89,855 Asian women (mean age: 43.5 ± 8.7 years; mean BMI: 22.3 ± 3.1 kg/m^2^) who underwent initial mammographic examinations for breast cancer screening were included in the analysis (Fig. 1). Our study cohort included 143 breast cancers (0.16%, 143/89,855). Table 1 presents the baseline characteristics of the participants categorized according to their registered breast cancer status. The majority of participants exhibited either heterogeneously or extremely dense breasts according to both radiologists’ readings and AI-based results (87.1% and 80.8%, respectively). Women with breast cancer were more likely to be older, postmenopausal, and obese compared with women without breast cancer.

**Table 1:** Baseline characteristics of the entire study population

	All women (*n* = 89,855)	No breast cancer (*n* = 89,712)	Breast cancer (*n* = 143)
Age (years)^*^	43.5 (8.7)	43.5 (8.7)	46.6 (9.2)
Menopausal status			
Premenopausal	72,620 (80.8)	72,512 (80.8)	108 (75.5)
Postmenopausal	16,953 (18.9)	16,920 (18.9)	33 (23.1)
Missing	282 (0.3)	280 (0.3)	2 (1.4)
Body mass index category			
Underweight	6,603 (7.4)	6,596 (7.4)	7 (4.9)
Normal	51,990 (57.9)	51,913 (57.9)	77 (53.9)
Overweight	15,763 (17.5)	15,742 (17.6)	21 (14.7)
Obese	15,395 (17.1)	15,358 (17.1)	37 (25.9)
Missing	104 (0.1)	103 (0.1)	1 (0.7)
Education level			
< College graduate	26,018 (29.0)	25,972 (29.0)	46 (32.2)
≥ College graduate	58,191 (64.8)	58,105 (64.8)	86 (60.1)
Unknown	5,646 (6.3)	5,635 (6.3)	11 (7.7)
First-degree family history of breast cancer			
Yes	2,410 (2.7)	2,407 (2.7)	3 (2.1)
No	87,171 (97.0)	87,032 (97.0)	139 (97.2)
Unknown	274 (0.3)	273 (0.3)	1 (0.7)
History of benign breast disease^†^			
Yes	7,885 (8.8)	7,867 (8.8)	18 (12.6)
No	74,141 (82.5)	74,029 (82.5)	112 (78.3)
Unknown	7,829 (8.7)	7,816 (8.7)	13 (9.1)
Five-year risk based on Gail model (%)			
< 0.83%	88,972 (99.0)	88,831 (99.0)	141 (98.6)
0.83–1.66%	593 (0.7)	593 (0.7)	0 (0)
≥ 1.67%	5 (0.0)	5 (0.0)	0 (0)
Unknown	285 (0.3)	283 (0.3)	2 (1.4)
Equipment (%)			
Senographe, GE	87,686 (97.6)	87,545 (97.6)	141 (98.6)
Selenia, Hologic	2,169 (2.4)	2,167 (2.4)	2 (1.4)
Mammography density			
A. Almost entirely fatty	1,035 (1.2)	1,033 (1.2)	2 (1.4)
B. Scattered fibroglandular tissue	10,528 (11.7)	10,512 (11.7)	16 11.2)
C. Heterogeneously dense	40,943 (45.6)	40,877 (45.6)	66 (46.2)
D. Extremely dense	37,349 (41.6)	37,290 (41.6)	59 (41.3)
AI-driven mammographic density			
A. Almost entirely fatty	501 (0.6)	500 (0.6)	1 (0.7)
B. Scattered fibroglandular tissue	16,771 (18.7)	16,748 (18.7)	23 16.1)
C. Heterogeneously dense	55,947 (62.3)	55,856 (62.3)	91 63.6)
D. Extremely dense	16,636 (18.5)	16,608 (18.5)	28 (19.6)

*Note*: Unless otherwise specified, data are presented as numbers of participants, with percentages in parentheses

^*^Data are presented as means with standard deviation in parentheses

^†^History of benign breast disease was collected starting from 2011; therefore, data on this aspect were unavailable for women who underwent screening in 2009 and 2010

### Performance analysis of screening mammography by radiologists and AI algorithm

Table 2 presents the performance analysis results of screening mammography for both radiologists and the AI algorithm. The CDR was 1.1 (95% CI, 0.9–1.4) per 1000 examinations for radiologists and 1.1 (95% CI, 0.9–1.13) for the AI algorithm. The sensitivity was slightly higher for radiologists (69.9% [95% CI, 61.7–77.3]) than that of the AI algorithm (67.1% [95% CI, 58.8–74.8]), although the difference was not statistically significant (*P* = 0.516). Meanwhile, other indices favored the AI algorithm over radiologists. The specificity was higher for the AI algorithm at 93.0% (95% CI, 92.9–93.2%), compared with 77.6% (95% CI, 61.7–77.9%) for radiologists (*P* < 0.001). The PPV was also higher for the AI algorithm at 1.5% (95% CI, 1.2–1.9%) versus 0.5% (95% CI, 0.4–0.6%) for radiologists (*P* < 0.001). Additionally, the AUC value for the AI algorithm was 0.80 (95% CI, 0.76–0.84), compared with 0.74 (95% CI, 0.7–0.78) for radiologists (*P* = 0.004). The recall rate was three times lower for the AI algorithm (7.1% [95% CI, 6.9–7.2]), which differed significantly from that of radiologists (22.5% [95% CI, 22.2–22.7]; *P* = 0.004).

**Table 2:** Performance of screening mammography compared between radiologists and standalone AI

Outcome	Radiologists’ BI-RADS category (0, 3, 4, 5)		Standalone AI (Cutoff 10%)		P value
	Estimate	95% CI	Estimate	95% CI	
CDR, per 1000 examinations	1.1	0.9–1.4	1.1	0.9–1.3	0.516
Sensitivity, %	69.9	61.7–77.3	67.1	58.8–74.8	0.516
Specificity, %	77.6	61.7–77.9	93.0	92.9–93.2	< 0.001
PPV, %	0.5	0.4–0.6	1.5	1.2–1.9	< 0.001
Recall rate, %	22.5	22.2–22.7	7.1	6.9–7.2	< 0.001
AUC	0.74	0.70–0.78	0.80	0.76–0.84	0.004

AI, artificial intelligence; AUC, area under the receiver operating characteristic curve; BI-RADS, Breast Imaging Reporting and Data System; CDR, cancer detection rate; CI, confidence interval; PPV, positive predictive value

In a sensitivity analysis focused on women aged 40 and above, the recommended demographic for mammographic screening in Korea, a similar trend was noted. Standalone AI outperformed radiologists in specificity, PPV, and recall rate, but fell short in CDR and sensitivity (Additional file 1: Table S1).

### Subgroup analyses by breast density category based on radiologist reports and AI algorithm results

Table 3 presents the performance metrics of screening mammography by breast density category based on radiologist reports. Both radiologists and the AI algorithm showed the best performance metrics for non-dense breasts. In non-dense breast category, the CDR and sensitivity were the same for both radiologists and the AI algorithm, with 1.2 per 1000 examinations (95% CI, 0.7–2.0) and 77.8% (95% CI, 52.4–93.6%), respectively. Specificity, PPV, and recall rate were more favorable for the AI algorithm compared with radiologists (specificity, 96.1% versus 86.5%; PPV, 3.0% versus 0.9%; recall rate, 4.0% versus 13.6%; all *P* < 0.001). AUC values tended to be higher for the AI algorithm (0.87 [95% CI, 0.77–0.97] vs. 0.82 [95% CI, 0.72–0.92]), although this difference did not reach statistical significance (*P* = 0.234). In the heterogeneously dense breast category, radiologists showed a tendency towards a higher CDR and sensitivity without statistical significance (CDR, 1.2 versus 1.0 per 1000 examinations; sensitivity, 75.8% versus 63.6%; *P* = 0.059). Meanwhile, the AI algorithm consistently outperformed radiologist in terms of specificity, PPV, and recall rate (specificity, 93.6% versus 77.9%; PPV, 1.6% versus 0.6%; recall rate; 6.5% versus 22.2%; all *P* < 0.001). In the extremely dense breast category, all performance metrics favored the AI algorithm, with significant improvements in specificity, PPV, and recall rate compared with radiologists (specificity, 91.5% versus 74.5%; PPV, 1.2% versus 0.4%; recall rate, 8.6% versus 25.5%; all *P* < 0.001). However, no significant differences were observed in the CDR, sensitivity, or AUC value between the two groups. Notably, the recall rates of the AI algorithm were approximately one-third of those achieved by radiologists across all breast density categories.

**Table 3:** Performance of screening mammography compared between radiologists and standalone AI by BI-RADS breast density category

Outcome	Radiologists’ BI-RADS category (0, 3, 4, 5)		Standalone AI (Cutoff 10%)		P value
	Estimate	95% CI	Estimate	95% CI	
**Non-dense**					
CDR, per 1000 examinations	1.2	0.7–2.0	1.2	0.7–2.0	1.000
Sensitivity, %	77.8	52.4–93.6	77.8	52.4–93.6	1.000
Specificity, %	86.5	85.9–87.1	96.1	95.8–96.5	< 0.001
PPV, %	0.9	0.5–1.5	3.0	1.7–5.1	< 0.001
Recall rate, %	13.6	13.0–14.2	4.0	3.6–4.4	< 0.001
AUC	0.82	0.72–0.92	0.87	0.77–0.97	0.234
**Heterogeneously dense**					
CDR, per 1000 examinations	1.2	0.9–1.6	1.0	0.8–1.4	0.059
Sensitivity, %	75.8	63.6–85.5	63.6	50.9–75.1	0.059
Specificity, %	77.9	77.5–78.3	93.6	93.4–93.8	< 0.001
PPV, %	0.6	0.4–0.7	1.6	1.1–2.1	< 0.001
Recall rate, %	22.2	21.8–22.6	6.5	6.3–6.7	< 0.001
AUC	0.77	0.72–0.82	0.79	0.73–0.85	0.575
**Extremely dense**					
CDR, per 1000 examinations	1.0	0.7–1.3	1.1	1.1–1.5	0.346
Sensitivity, %	61.0	47.4–73.5	67.8	54.4–79.4	0.346
Specificity, %	74.5	74.1–75.0	91.5	91.2–91.7	< 0.001
PPV, %	0.4	0.3–0.5	1.2	0.9–1.7	< 0.001
Recall rate, %	25.5	25.1–26.0	8.6	8.4–8.9	< 0.001
AUC	0.68	0.62–0.74	0.80	0.74–0.86	0.297

AI, artificial intelligence; AUC, area under the receiver operating characteristic curve; BI-RADS, Breast Imaging Reporting and Data System; CDR, cancer detection rate; CI, confidence interval; PPV, positive predictive value

Similar patterns were observed when using breast density category based on the AI algorithm instead of radiologist reports (Table 4). Although the CDR and sensitivity did not exhibit significant differences between AI and radiologists, the AI algorithm demonstrated superior performance in terms of specificity, PPV, and recall rates. Notably, the AI algorithm consistently achieved significantly lower recall rates compared to those attained by radiologists across all breast density categories. In extremely dense breasts, the AI algorithm outperformed in all performance metrics, with statistical significance observed for specificity, PPV, recall rate, and AUC metrics.

**Table 4:** Performance of screening mammography compared between radiologists and standalone AI according to AI-based breast density

Outcome	Radiologists’ BI-RADS category (0, 3, 4, 5)		Standalone AI (Cutoff 10%)		P value
	Estimate	95% CI	Estimate	95% CI	
**Non-dense (A or B)**					
CDR, per 1000 examinations	1.2	0.7–1.8	1.0	0.7–1.7	0.317
Sensitivity, %	83.3	62.6–95.3	75	53.3–90.2	0.317
Specificity, %	78.8	78.1–79.4	96.3	96.0–96.6	< 0.001
PPV, %	0.5	0.3–0.8	2.8	1.6–4.3	< 0.001
Recall rate, %	21.3	20.7–22.0	3.8	3.5–4.1	< 0.001
AUC	0.81	0.73–0.89	0.86	0.77–0.95	0.268
**Heterogeneously dense (C)**					
CDR, per 1000 examinations	1.1	0.9–1.4	1.0	0.8–1.3	0.257
Sensitivity, %	69.2	58.7–78.5	62.6	51.9–72.6	0.257
Specificity, %	77.1	76.7–77.4	93.2	92.9–93.4	< 0.001
PPV, %	0.5	0.4–0.6	1.5	1.1–1.9	< 0.001
Recall rate, %	23.0	22.7–23.4	6.9	6.7–7.2	< 0.001
AUC	0.73	0.68–0.78	0.78	0.73–0.83	0.103
**Extremely dense (D)**					
CDR, per 1000 examinations	1	0.6–1.6	1.3	0.8–1.9	0.103
Sensitivity, %	60.7	40.6–78.5	75.0	55.1–89.3	0.103
Specificity, %	78.3	77.7–78.9	89.2	88.8–89.7	< 0.001
PPV, %	0.5	0.3–0.8	1.2	0.7–1.8	0.004
Recall rate, %	21.8	21.1–22.4	10.9	10.4–11.3	< 0.001
AUC	0.70	0.60–0.79	0.82	0.74–0.90	0.003

AI, artificial intelligence; AUC, area under the receiver operating characteristic curve; BI-RADS, Breast Imaging Reporting and Data System; CDR, cancer detection rate; CI, confidence interval; PPV, positive predictive value

### Characteristics of positive breast cancer cases by radiologists and AI algorithm

Table 5 presents the characteristics of 143 breast cancers identified in the national cancer registry data within 12 months of mammographic screening. Among all patients, 35 (24.5%) ductal carcinomas in situ and 108 (75.5%) invasive cancers were identified. The majority of breast cancers were localized cancers (108/143, 75.5%), followed by regional cancers (31/143, 21.7%), and distant metastasis (1 case, 0.7%). Among the 143 cancers, 100 were detected by radiologists and 96 were detected by the AI algorithm. Among positive cancers, 79 (55.2%) were detected by both radiologists and the AI algorithm, 21 (14.7%) were detected by radiologists only, and 17 (11.1%) were detected by AI only (Additional file 1: Table S2). Cancers detected by the AI algorithm were more invasive (73/96, 76%) compared with those detected by radiologists (69/100, 69%) (*P* = 0.038). The proportion of cancers in the regional stage was higher in cancers detected by the AI algorithm (27.1% [26/96] vs. 20% [20/100]), and the proportion of localized cancers was higher in cancers detected by radiologists (78.0% [78/100] versus 70.8% [68/96]), but failed to get statistical significance (all *P* > 0.05). The AI algorithm detected 41.7% (40/96) of cancers in the extremely dense breast category compared with 36.0% (36/100) detected by radiologists. The time intervals from the screening mammography to cancer diagnosis were similar between positive cases identified by radiologists and those identified by the AI algorithm, with a median of 1.54 months. However, the interval was slightly longer for all breast cancer cases, with a median interval of 2.46 months (interquartile ranges, 0.95–9.26).

**Table 5:** Characteristics of 143 breast cancers

	All cancers	Radiologist-positive cancers	AI-positive cancers	P value
Total number of cancers	143	100	96	0.610
Age (years)	46.6 (9.2)	47.8 (9.5)	46.5 (9.6)	0.096
Cancer type				
Ductal carcinoma in situ	35 (24.5)	31 (31.0)	23 (23.9)	0.038
Invasive	108 (75.5)	69 (69.0)	73 (76.0)	0.038
SEER				
Localized	108 (75.5)	78 (78.0)	68 (70.8)	0.577
Regional	31 (21.7)	20 (20.0)	26 (27.1)	0.284
Distant	1 (0.7)	0 (0.0)	0 (0.0)	-
Unknown	3 (2.1)	2 (2.0)	2 (2.1)	0.921
Treatment modality				
Surgery	129 (90.2)	90 (90.0)	84 (87.5)	0.199
Chemotherapy	52 (36.4)	31 (31.0)	38 (39.6)	0.515
Radiotherapy	38 (26.6)	24 (24.0)	21 (21.9)	0.049
Hormone therapy	32 (22.4)	19 (19.0)	18 (18.8)	0.044
No treatment	4 (2.8)	3 (3.0)	3 (3.1)	0.672
Time to cancer diagnosis since screening mammography (months)^*^	2.46 (0.95–9.26)	1.54 (0.69–5.21)	1.54 (0.66–5.17)	< 0.001
Mammographic density				
Non-dense	18 (12.6)	14 (14.0)	14 (14.6)	0.164
Dense	125 (87.4)	86 (86.0)	82 (85.4)	0.164
Mammography density				
Almost entirely fatty	2 (1.4)	1 (1.0)	1 (1.0)	0.455
Scattered fibroglandular tissue	16 (11.2)	13 (13.0)	13 (13.5)	0.058
Heterogeneously dense	66 (46.2)	50 (50.0)	42 (43.8)	0.663
Extremely dense	59 (41.3)	36 (36.0)	40 (41.7)	0.198
Mammographic density-AI				
A	1 (0.7)	1 (1.0)	1 (1.0)	-
B	23 (16.1)	19 (19.0)	17 (17.7)	0.082
C	91 (63.6)	63 (63.0)	57 (59.4)	0.210
D	28 (19.6)	17 (17.0)	32 (21.9)	0.876

*Note*: Unless otherwise specified, data are presented as numbers of participants, with percentages in parentheses

SEER, Surveillance, Epidemiology, and End Results

^*^Data are presented as means, with interquartile ranges in parentheses

## Discussion

We investigated the performance metrics of initial screening mammography using a standalone AI algorithm compared with those of radiologists among Asian women, considering breast density. Overall, the CDR and sensitivity were similar between radiologists and the AI algorithm. However, the AI algorithm outperformed radiologists in terms of specificity, PPV, recall rate, and AUC value. A subgroup analysis based on breast density revealed that the sensitivity and CDR tended to be lower for the AI algorithm in heterogeneously dense breasts. In contrast, the AI algorithm showed better performance in extremely dense breasts, although the CDR and sensitivity showed no significant differences between radiologists and the AI algorithm. The specificity, PPV, and recall rate consistently favored the AI algorithm across all breast density categories.

Previous retrospective studies have reported that AI support helps radiologists improve diagnostic accuracy in both reader studies using cancer-enriched dataset and external validation studies using real-world screening mammograms [10–12, 16, 17]. Moreover, incorporating AI systems into the reading protocol of population-based breast cancer screening programs has demonstrated the potential to reduce radiologists’ workload without compromising diagnostic performance [24, 37]. Notably, a recent prospective, population-based reader study demonstrated that double reading by one radiologist plus AI resulted in an increased CDR by 4% compared with standard double reading by two radiologists [38]. In addition, findings from a randomised, controlled, population-based trial indicated that AI-supported mammography screening resulted in a similar CDR while substantially reducing the screen-reading workload compared with standard double reading [39]. In the assessment of standalone AI performance, a recent systemic review incorporating 13 studies on digital mammography revealed significantly higher AUCs for standalone AI compared to radiologists in six reader studies involving cancer-enriched populations. However, this improvement was not observed in seven historic cohort studies, demonstrating higher sensitivity and lower specificity irrespective of study type [18].

In our study, the standalone AI algorithm demonstrated significantly higher specificity, PPV, and AUC values compared with radiologists. Notably, the recall rate for the standalone AI was three times lower than that for radiologists, and this trend was consistent across breast density categories. Our findings indicate that the AI algorithm achieved a high level of accuracy, particularly by reducing the number of false-positive results and potentially enhancing the efficiency of mammography screening. The AI algorithm demonstrated the ability to detect invasive cancers and regional stage cancers more effectively than radiologists. Future research is warranted to ascertain whether AI can truly enhance the detection of prognostically poor cancers such as invasive cancers with node positivity.

Several prior studies explored AI performance in relation to breast density, noting a relative decline of standalone AI performance as breast density increases [40–42]. However, another study reported consistent sensitivity for an AI system with increased breast density, while radiologists’ sensitivity decreased [43]. In our study, performance metrics of the standalone AI were superior in women with non-dense breasts compared to dense breasts. Interestingly, the AI algorithm demonstrated superior performance in extremely dense breasts than heterogeneously dense breasts in terms of CDR, sensitivity, and AUC, along with increased detection of invasive cancers and regional stage cancers, despite showing inferior performance of specificity, PPV and recall rate. This performance pattern suggests that the AI algorithm could serve as a valuable complementary tool to reduce the risk of overlooking advanced cancer cases, particularly in patients with extremely dense breast tissue.

The CDR and sensitivity were not significantly different between standalone AI and radiologists, which differs from previous studies. Although we used AI algorithm which was developed and validated with mammograms from both Asian and Western population for precise evaluation in our screening cohort comprised of Korean women, both AI and radiologists exhibited low CDR and sensitivity. The low CDR observed in our study could be due to the relatively low incidence of breast cancer in this study population, with a rate of 0.16% (143/89,555) compared to previous historic cohort studies (0.7 to 3.4%) [15, 18, 23–26]. The exclusion of women who received supplementary breast ultrasound, particularly those with mammographically dense breasts or high-risk factors, might have contributed to the low observed breast cancer rate. Additionally, we only included the first mammograms during the study, resulting in a relatively young study population with a mean age of 43.5 ± 8.7 years. The high proportion of women under 40 years old may contribute to the low breast cancer rate in our study cohort. The limited sensitivity could be an inherent weakness of mammography in Asian women with small dense breasts owing to the masking effect of the surrounding fibroglandular tissue, rather than the inferior performance of either radiologists or AI. It is noted that the percent of the breast occupied by dense tissue is higher in Asian women than Caucasian women [44]. Our study population’s breast area was nearly half that of Black women (90.3 cm^2^ versus 180.5 cm^2^) and 50–69% of White women (130–155 cm^2^), while the dense area observed in our study was slightly higher than that reported for the Western population (27.1 cm^2^ versus 22.3–25.9 cm^2^), and the breast density was higher (33.2% versus 14.9–17.1%) [45, 46] (Additional file: Tables S3 and S4). In fact, the majority of women (87.1%) had dense breasts in our study; 41.6% had extremely dense breasts and 45.6% had heterogeneously dense breasts according to the BI-RADS. Specifically, our study showed a sensitivity of 69.9%, and specificity of 77.6%, all of which were inferior to the Breast Cancer Surveillance Consortium mammography screening benchmarks (sensitivity, 87.6%; specificity, 90.2%) [47]. For dense breasts, our performance was lower compared to results from the U.S. Breast Cancer Surveillance Consortium (sensitivity, 61.0–75.8% versus 72.6–82.4%; specificity, 74.5–77.9% versus 90.1–91.0%) [48]. However, our results were better or comparable to large-scale analyses for over 8 million Korean women (sensitivities for dense breasts, 62.0–74.8%; specificities, 71.4–82.5%) [5, 49]. Therefore, the limited performance observed in our study could potentially be attributed to the unique characteristics of our study population, consisting of young Asian women, with higher dense area, greater breast density, and smaller breast size compared to the Western population. Further research is warranted to explore the relationship between breast size, density, and mammographic performance more comprehensively.

In our study, the recall rate of the radiologists was high (22.5%) compared to both the American College of Radiology BI-RADS atlas and the Breast Cancer Surveillance Consortium mammography screening benchmarks (5–12%) [32, 47]. This high recall rate could be attributed to our study’s specific focus on the initial mammograms during the study period. Previous research has shown that recall rates for first-time mammograms are significantly higher, by approximately 50% compared to those for subsequent mammograms [50]. Our result was similar to the 21.3% recall rates reported for baseline mammograms from the Breast Cancer Surveillance Consortium registries [51]. In addition, when we extended our analysis to include all first and subsequent mammograms within the study period (*n* = 182,926), the recall rate decreased to 11.0% (95% CI, 10.9–11.2%), which falls within the acceptable range by mammography screening benchmarks.

Our study had several limitations that warrant careful consideration when interpreting the results. First, the study population comprised women who participated in private screening programs at a single tertiary hospital. As the proportion of young women with dense breasts was relatively high and the participants were predominantly employees of various companies and local governmental organizations and their spouses. Consequently, the participants were mostly well-educated individuals with high accessibility to medical services. Also, we only included women who underwent first digital mammography without supplementary breast ultrasound, resulting in low observed cancer rates and high recall rates. These could limit the generalizability of our findings to a broader population. Second, while we recommended supplementary ultrasonography for women with dense breasts, the data used in our analysis relied solely on screening tests conducted at the health promotion center without access to other medical records beyond the screening examination. Acknowledging that some participants may have been referred for additional examinations, such as breast ultrasonography, is essential as this could have influenced the detection of additional breast cancer. Despite our efforts to evaluate the screening performance of mammography while excluding other supplementary tests, the possibility of additional unmeasured tests affecting the breast cancer diagnosis remains a potential confounding factor. Third, our analysis was based on retrospective data collected during routine health examinations and previous radiologic screening mammography reports. Therefore, we did not assess the utility of the AI algorithm for radiologists in a real screening setting, nor did we evaluate its potential impact on screening performance when used by radiologists. Further prospective studies are required to comprehensively understand the effectiveness of the AI algorithm in real-world screening environments. Fourth, the diagnostic performance may have been influenced by interobserver variability among the radiologists interpreting the mammograms. However, our retrospective analysis, based on deidentified data without specific radiologist information, precluded accounting for this factor. Lastly, we did not directly assess the impact of various characteristics such as geographic location, age, race, ethnicity, breast size, and density distribution on diagnostic performance. Further research is needed to explore these aspects and to comprehensively understand their impact on diagnostic accuracy.

## Conclusions

In a large group of Korean women, standalone AI showed superior performance over radiologists in terms of specificity, PPV, recall rate, and AUC. The most significant differences were observed in cases of extremely dense breast tissue, while no notable distinctions emerged in CDR and sensitivity. The results underscore the AI algorithm’s heightened accuracy relative to radiologists, particularly in reducing false positives and identifying invasive cancers, especially in cases of extremely dense breasts. These findings underscore the potential of AI algorithms to improve the effectiveness of breast cancer screening for Asian women. However, future prospective studies, including diverse populations and an evaluation of the AI algorithm’s impact in a screening context, are necessary to validate and deepen our understanding of its effectiveness.

### Electronic supplementary material

Below is the link to the electronic supplementary material.

Supplementary Material 1

## Data Availability

The datasets generated during and/or analyzed during the current study are not publicly available due to Institutional Review Board restrictions (the data were not collected in a way that could be distributed widely) but are available from the corresponding author on reasonable request.

## Abbreviations

AI: Artificial intelligence
AUC: Area under the receiver operating characteristic curve
BI-RADS: Breast Imaging Reporting and Data System
BMI: Body mass index
CAD: Computer-aided diagnosis
CC: Craniocaudal
CDR: Cancer detection rate
CI: Confidence interval
MLO: Mediolateral oblique
PPV: Positive predictive value
