# Supplementary material for: Screening mammography performance according to breast density: a comparison between radiologists versus standalone intelligence detection
Source: Breast Cancer Res. 2024 Apr 22;26:68. doi: 10.1186/s13058-024-01821-w (PMC11036604; doi:10.1186/s13058-024-01821-w)
Supplement: Supplementary file 1 — Supplementary Material 1 [file 13058_2024_1821_MOESM1_ESM.docx]

**Supplementary Files**

Table S1. Performance of screening mammography compared between radiologists and standalone AI among women ≥ 40 years

| Outcome | Radiologists’  BI-RADS category (0, 3, 4, 5) | | Standalone AI  (Cutoff 10%) | | P value |
| --- | --- | --- | --- | --- | --- |
|  | Estimate | 95% CI | Estimate | 95% CI |  |
| CDR, per 1000 examinations | 1.6 | 1.3–2.0 | 1.4 | 1.1–1.8 | 0.041 |
| Sensitivity, % | 73.1 | 63.8–81.2 | 63.0 | 53.1–72.1 | 0.041 |
| Specificity, % | 78.3 | 78.0–78.7 | 93.5 | 93.3–93.7 | <0.001 |
| PPV, % | 0.7 | 0.6–0.9 | 2.1 | 1.6–2.6 | <0.001 |
| Recall rate, % | 21.8 | 21.4–22.1 | 6.6 | 6.4–6.8 | <0.001 |
| AUC | 0.76 | 0.72–0.80 | 0.78 | 0.74–0.83 | 0.310 |

Table S2. Cancer characteristics according to detection method

|  | Cancers detected by both | Cancers detected by radiologists only | Cancers detected by AI only | Cancers not detected by both |
| --- | --- | --- | --- | --- |
| Total number of cancers | 79 | 21 | 17 | 26 |
| Age (years) | 47.9 (9.9) | 47.8 (8.2) | 40.3 (4.4) | 46.2 (8.9) |
| Cancer type |  |  |  |  |
| Ductal carcinoma in situ | 22 (27.9) | 9 (42.9) | 1 (5.9) | 3 (11.5) |
| Invasive | 57 (72.5) | 12 (57.1) | 16 (94.1) | 23 (88.5) |
| SEER |  |  |  |  |
| Localized | 58 (73.4) | 20 (95.2) | 10 (58.8) | 20 (76.9) |
| Regional | 19 (24.1) | 1 (4.8) | 7 (41.2) | 4 (15.4) |
| Distant | 0 (0.0) | 0 (0.0) | 0 (0.0) | 1 (3.9) |
| Unknown | 2 (2.5) | 0 (0.0) | 0 (0.0) | 1 (3.9) |
| Treatment modality |  |  |  |  |
| Surgery | 70 (88.6) | 20 (95.2) | 14 (82.4) | 25 (96.2) |
| Chemotherapy | 28 (35.4) | 3 (14.3) | 10 (58.8) | 11 (42.3) |
| Radiotherapy | 20 (25.3) | 4 (19.1) | 1 (5.9) | 13 (50.0) |
| Hormone therapy | 15 (19.0) | 4 (19.1) | 3 (17.7) | 10 (38.5) |
| No treatment | 2 (2.5) | 1 (4.8) | 1 (5.9) | 0 (0.0) |
| Time to cancer diagnosis since screening mammography (months)^*2^ | 1.22 (0.59–3.91) | 5.32 (1.77–10.09) | 9.69 (3.29–11.43) | 9.26 (5.42–9.86) |
| Mammographic density |  |  |  |  |
| Non-dense | 13 (16.5) | 1 (4.8) | 1 (5.9) | 3 (11.5) |
| Dense | 66 (83.5) | 20 (95.2) | 16 (94.1) | 23 (88.5) |
| Mammography density |  |  |  |  |
| Almost entirely fatty | 1 (1.3) | 0 (0.0) | 0 (0.0) | 1 (3.9) |
| Scattered fibroglandular tissue | 12 (15.2) | 1 (4.8) | 1 (5.9) | 2 (7.7) |
| Heterogeneously dense | 37 (46.8) | 13 (61.9) | 5 (29.4) | 11 (42.3) |
| Extremely dense | 29 (36.7) | 7 (33.3) | 11 (64.7) | 12 (46.2) |
| Mammographic density-AI |  |  |  |  |
| A | 1 (1.4) | 0 (0.0) | 0 (0.0) | 0 (0.0) |
| B | 16 (20.3) | 3 (14.2) | 1 (5.9) | 3 (11.5) |
| C | 46 (58.2) | 17 (81.0) | 11 (64.7) | 17 (65.4) |
| D | 16 (20.3) | 1 (4.8) | 5 (29.4) | 6 (23.1) |

Table S3. Comparison of breast area, dense area, and percent density of study population using LIBRA software with previous research

|  | Study population  (n=89804^*^) | Mostly white women with breast cancer^†^ (n=437) | Mostly white women without breast cancer^†^ (n=1225) | White women^‡^  (n=4216) | Black women^‡^  (n=5282) |
| --- | --- | --- | --- | --- | --- |
|  | Mean (SD) | Median | Median | Mean | Mean |
| Breast area, cm^2^ | 90.3 (30.5) | 154.2^§^ | 155.0^§^ | 130.4^§^ | 180.5^§^ |
| Dense area, cm^2^ | 27.1 (19.9) | 25.9 | 23.1 | 22.3 | 22.2 |
| Area percent density | 33.2 (22.1) | 16.8 | 14.9 | 17.1 | 12.3 |

^*^51 mammograms of 89855 participants failed to be inferred using LIBRA software

^†^Data from Gastounioti et al. (2020) [S1]

‡Data from McCarthy et al. (2016) [S2]

^§^ Based on percent density and dense area, estimated breast area is calculated.

Table S4. LIBRA-based breast area, percent density and dense areas of 89804 mammograms

|  | Mean | SD | Min | p10 | p25 | p50 | p75 | p90 | Max |
| --- | --- | --- | --- | --- | --- | --- | --- | --- | --- |
| Breast area, cm^2^ | 90.25713 | 30.48025 | 17.9 | 56.0 | 68.6 | 85.5 | 106.9 | 130.5 | 317.6 |
| Breast area, CC view, cm^2^ | 83.16413 | 30.93926 | 16.1 | 49.1 | 61.1 | 77.8 | 99.7 | 124.1 | 322.3 |
| Breast area, MLO view, cm^2^ | 97.3502 | 31.32363 | 17.1 | 61.1 | 75.4 | 93.5 | 115.0 | 138.3 | 325.6 |
| Percent density, % | 33.23032 | 22.09351 | 0.8 | 4.8 | 13.9 | 31.2 | 50.3 | 64.3 | 99.0 |
| Percent density, CC, % | 35.97766 | 23.7698 | 0.7 | 4.3 | 14.5 | 34.7 | 55.4 | 68.6 | 99.2 |
| Percent density, MLO, % | 30.48299 | 21.52293 | 0.6 | 4.6 | 12.3 | 27.0 | 45.2 | 61.5 | 99.4 |
| Dense area, cm^2^ | 27.12445 | 19.8704 | 1.1 | 4.9 | 12.1 | 23.8 | 37.4 | 52.4 | 228.2 |
| Dense area, CC, cm^2^ | 27.06702 | 19.91829 | 1.0 | 4.1 | 11.8 | 24.3 | 37.6 | 52.2 | 241.4 |
| Dense area, MLO, cm^2^ | 27.18188 | 20.79122 | 1.0 | 5.1 | 11.7 | 22.9 | 37.3 | 54.1 | 224.8 |

^*^51 mammograms of 89855 participants failed to be inferred using LIBRA software

LIBRA, an automated, open-source software, is designed for both raw and processed FFDM images to calculate mammographic breast density [S3]. It detects the breast boundary using edge-detection algorithms and segments it into dense and non-dense areas through fuzzy c-means clustering based on gray-level intensity. LIBRA calculates the dense area and normalizes this by the total breast area to determine the percent density (PD). In this analysis, dense area, breast area and percent density metrics were obtained from LIBRA for each woman's stored processed images. Averages were taken across all four standard mammographic views—bilateral craniocaudal (CC) and mediolateral oblique (MLO) views [S2]. Additionally, averages were specifically calculated for bilateral CC views and bilateral MLO views separately.

**Supplementary References:**

**S1.** Gastounioti A, Kasi CD, Scott CG, Brandt KR, Jensen MR, Hruska CB, Wu FF, Norman AD, Conant EF, Winham SJ et al: Evaluation of LIBRA Software for Fully Automated Mammographic Density Assessment in Breast Cancer Risk Prediction. Radiology 2020, 296(1):24-31.

**S2.** McCarthy AM, Keller BM, Pantalone LM, Hsieh MK, Synnestvedt M, Conant EF, Armstrong K, Kontos D: Racial Differences in Quantitative Measures of Area and Volumetric Breast Density. J Natl Cancer Inst 2016, 108(10).

**S3.** Keller BM, Chen J, Daye D, Conant EF, Kontos D: Preliminary evaluation of the publicly available Laboratory for Breast Radiodensity Assessment (LIBRA) software tool: comparison of fully automated area and volumetric density measures in a case-control study with digital mammography. Breast Cancer Res 2015, 17:117.
